# How to assess the structural dynamics of transcription factors by integrating sparse NMR and EPR constraints with molecular dynamics simulations

**DOI:** 10.1016/j.csbj.2021.04.020

**Published:** 2021-04-21

**Authors:** Fanny Kozak, Dennis Kurzbach

**Affiliations:** University Vienna, Faculty of Chemistry, Institute of Biological Chemistry, Waehringer Str. 38, 1090 Vienna, Austria

**Keywords:** NMR, EPR, MD simulations, Transcription factors, Structural dynamics

## Abstract

We review recent advances in modeling structural ensembles of transcription factors from nuclear magnetic resonance (NMR) and electron paramagnetic resonance (EPR) spectroscopic data, integrated with molecular dynamics (MD) simulations. We focus on approaches that confirm computed conformational ensembles by sparse constraints obtained from magnetic resonance. This combination enables the deduction of functional and structural protein models even if nuclear Overhauser effects (NOEs) are too scarce for conventional structure determination. We highlight recent insights into the folding-upon-DNA binding transitions of intrinsically disordered transcription factors that could be assessed using such integrative approaches.

## Highlights

• Integrative structural biology of intrinsically disordered proteins.
• Combined studies of NMR, EPR and MD simulations of transcription factors.
• Spectroscopic and computational insights into DNA-interactions of intrinsically disordered transcription factors.

## Graphical abstract

**Figure:**
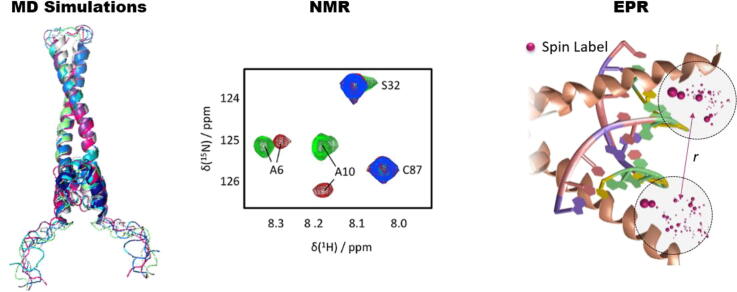

## 1 Introduction

Nuclear magnetic resonance (NMR) spectroscopy is a versatile tool encountered in many research fields.[1], [2], [3] In particular, for assessing protein structure and functionality in solution, NMR has evolved into a key technology as it provides access to dynamic parameters at atomistic resolution.[4], [5], [6], [7] In the past decade, this potential was especially evidenced at the example of the so-called intrinsically disordered regions (IDR) and proteins (IDPs).[8], [9], [10], [11], [12] These display high degrees of structural plasticity and consequently also complicated patterns of conformational sampling. As a result, their structural characterization remains challenging, not least because X-ray crystallography is not applicable, and novel approaches had to be developed.[13], [14], [15]

The sparseness of structural constraints based on nuclear Overhauser enhancements (NOEs) poses a significant bottleneck in the characterization of IDRs and IDPs. Indeed, established approaches [16], [17], [18] to structure calculation cannot be applied since these have been mainly developed for well-folded proteins that feature long-lived secondary and tertiary structural elements. Computational approaches based on molecular dynamics (MD) simulations are valuable complements to spectroscopic data and can help overcome this bottleneck. These simulations can derive models even if only few experimental constraints are available[19], [20], [21], [22], [23], [24], [25]. Concerning structural biology, a large share of applications of MD simulations is based on the assessment of time-resolved protein structural dynamics as well as protein–ligand interactions in either native aqueous or membrane environments[26], [27].

However, despite the exponentially growing power of modern computers, MD simulations often remain limited in the accuracy of their predictions and the quality of the employed force fields (FF) [28], [29]. Yet, therein lies the merit of the combination with NMR: while the use of MD simulations, as an additional source of information about the spatiotemporal configuration of a system, can assist the interpretation of NMR data, MD simulations can profit from the refinement of its predictions by spectroscopic constraints [30], [31], [32].

As a result, the combination of both methods allows for a description of biological macromolecules at a level of detail that would remain elusive to a single technique. In particular, the conformationally heterogenic conformational spaces of highly dynamic proteins can be depicted at high spatiotemporal resolution [33], [34].

Here, we aim at providing a brief overview of recent developments in applications of integrative magnetic resonance, i.e., NMR and electron paramagnetic resonance (EPR) spectroscopy, and molecular dynamics simulations with a particular focus on transcription factor (TF)-DNA interactions. We concentrate on intrinsically disordered TFs, i.e., on proteins that feature high degrees of structural plasticity either in their free or DNA-bound state, despite defined roles in cellular transcription. Besides, we highlight approaches that aim at resolving the dilemma between high affinities of TFs to their target DNA-motifs, despite nonetheless fast diffusion and dissociation along the double-strand.

The main principles behind the major magnetic resonance techniques, NMR and EPR spectroscopy, of IDPs, IDRs, and nucleic acids have been reviewed in detail, e.g. in references [35], [36], [37], [38].

For NMR spectroscopy, the target molecule is typically enriched either in ^15^N and/or ^13^C isotopes. A two- or higher-dimensional correlation spectrum is then recorded to resolve cross-peaks of individual residues (e.g., between ^1^H^N^ and ^15^N nuclei in an amino acid). The correlation spectra can be modified so that various parameters of the individual residues can be assessed. For example, longitudinal and transverse ^15^N-relaxation rates provide information about dynamics on the nanoseconds scale, or the secondary structure propensities can be derived from ^13^C secondary chemical shifts as a function of the primary sequence index [35]. A particular technique that relies on introducing a paramagnet in the IDP or IDR and that will be the focus of section 2 is to record residue-specific paramagnetic relaxation effects.

For EPR spectroscopy, the most frequent approach is to covalently attach a spin-label (SL) to the protein and directly detect the resonance of the unpaired electron. In the simplest case, the absorption of the electron is directly measured, which can provide information about the local dynamics of the SL [39]. If a second SL is introduced to the protein or nucleic acid simultaneously, the dipolar coupling [36], [40], [41] between the two SL can also be detected by so-called pulsed electron double resonance (PELDOR; also sometimes referred to as double electron–electron resonance: DEER) spectroscopy. The coupling depends on the proximity between the two electrons, such that distance distributions can be derived. This technique is the focus of section 3.

Finally, section 4 will highlight selected methodological considerations regarding the combination of NMR and EPR with MD simulations.

## 2 Combining NMR and MD simulations

In the past decades, many examples demonstrated the potential of NMR/MD combinations in determining structural features of transcription factors[42], [43], [44]. Recently, studies of intrinsically disordered transcription factors have experienced particular attention[45], [46], [47], [48]. This is not least a result of more powerful computers that provide longer MD trajectories and, hence, access to more complex conformational ensembles, [49] which is particularly useful for studying IDRs and IDPs[9], [50], [51], [52]. These proteins do not adopt stable secondary or tertiary structural elements but instead sample a continuum of continuously interconverting conformations.

Often, the DNA-binding domains of TFs display intrinsic disorder in solution. Only upon interaction with their target DNAs the binding regions fold into stable conformations. This process is often referred to as 'folding-upon-binding' or 'coupled folding and binding’ [53], [54]. Given the resulting complexity of the vast non-random-coil-like conformational and energy spaces, MD simulations are prone to finite-length effects. This risk can be mitigated by longer trajectories [55], [56], [57]. Besides, challenging simulations can be facilitated based on spectroscopic constraints that help to optimize force-field parameters (cf. section 4) [58], [59], [60].

*Integrating paramagnetic relaxation enhancements and MD simulations*

**Fig. 1:**
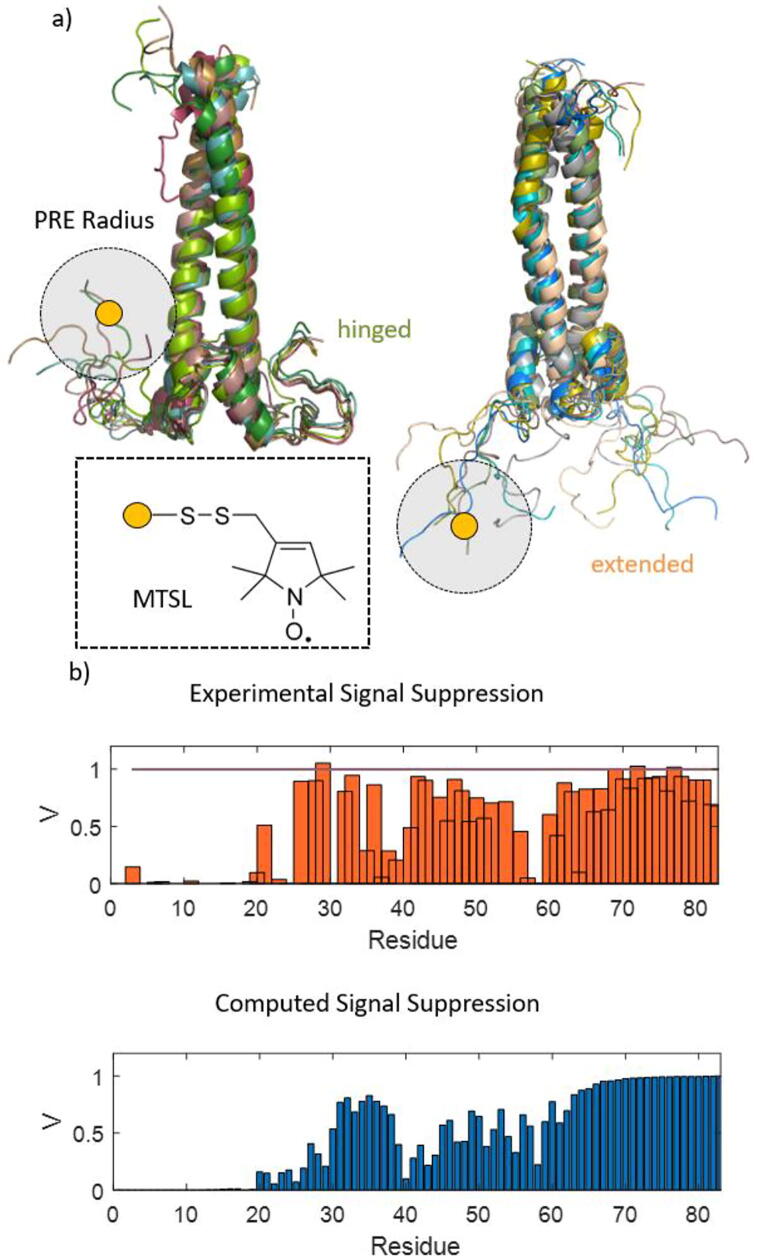
Comparison of experimental and computed PRE effects for the MAX transcription factor. a) Conformations sampled in MD simulations of the MAX:MAX homodimer. The DNA-binding domain (bottom) is intrinsically disordered in the absence of a ligand and samples a heterogeneous conformational space that includes hinged and extended conformations. If a spin-label is attached to the DNA-binding site (e.g. at the site of the yellow dot) NMR signals of amino acids in its vicinity are suppressed by PREs (effect range indicated by the grey shade). Thus, the hinged conformation would lead to the suppression of signals assigned to the remote HLH domain, while the extended conformations would not. The structure of the attached SL is indicated in the dashed box. b) Experimentally observed PRE effects as a function of residue position in MAX:MAX (top) compared to PRE effects extracted from MD simulations (bottom). The match between both data sets is good, such that the conformational ensemble sampled in the MD simulations could be verified by the experimental observations (adapted from reference [61] with permission of the publisher.) (For interpretation of the references to colour in this figure legend, the reader is referred to the web version of this article.)

Recently, approaches based on the site-specific introduction of paramagnetic labels could characterize the DNA-binding sites of (partially) disordered TFs[61], [62], [63], [64], [65]. These approaches employed the so-called paramagnetic relaxation enhancement (PRE) technique [66]. In the past years, NMR based on PREs has developed into a popular tool for elucidating IDR and IDP structural dynamics [67], [68]. PREs can reveal transient long-range contacts between different protein domains even if the contact time is short (ps-ns timescale). The protein is often site-selectively spin-labeled (SDSL) [39], [69], [70] with a paramagnetic tag, e.g., the nitroxide MTSL, covalently attached to a single amino acid. Residues in spatial proximity to the spin-label (SL) experience increased relaxation rates (*R*_1_ and *R*_2_), leading to line broadening and a reduction of signal amplitudes. The strength of this effect depends inversely on the distance *r* between the paramagnetic center and the NMR-detected nucleus by *r*^-6^ [66]. The distance information contained in this dependence can be used to infer structural models. The benefit of the combination with data from MD simulations lies in the potential to extract SL-to-residue distance constraints from computed trajectories and to predict the experimentally observed PRE effects (Fig. 1). The comparison of both data allows one to confirm (or discard) the computed conformational ensembles of the domain under study.

Combinations of PRE-NMR and MD simulations complement an extensive set of other approaches that combine NMR relaxometry and computation,[71], [72], [73] such that a complete review is beyond the scope of this article. Nevertheless, it is noteworthy that the rapid development of computing powers has also significantly extended this combination's scope in recent decades. While a few picoseconds-long trajectory had to suffice for Lipari and Szabo's [74] seminal work on the 'model-free’ approach, today, calculations beyond the millisecond scale are feasible on desktop computers [49]. Indeed, relaxation parameters bear the potential to elucidate structure–function relationships by assessing protein motions, and with the help of MD simulations, such information can be supplemented with structural models [75], [76].

Recently, putting the combination of PRE-based relaxometry and MD simulations to use, the intrinsically disordered DNA-binding interface of the important transcription factor MAX (MYC-associated factor X) has been investigated [61], [63], [77]. It could be shown that its conformational space is not merely void of long-lived structural elements. Instead, it contains a heterogeneous ensemble of states that enables rapid switching between extended and 'hinged' conformations. These findings complement long-standing research programs that aim at structure-elucidation of the DNA-binding sites of basic-helix-loop-helix-type transcription factors [78], [79], [80], [81], [82].

The reported switch likely facilitates DNA-TF-complex encounters by recruiting DNA to the host via extended conformations and exposing the DNA-binding site in the hinged state to foster the formation of the final conformation. Fig. 1 depicts the extended and hinged states. The figure also displays how PRE-NMR revealed the involved long-range contacts between the DNA-binding site and other domains of the MAX:MAX homodimer and how MD-simulations could predict these effects [61]. The combination of both data sets thus enabled the validation of the simulated conformational ensemble.

Based on a similar approach, the dynamics of the DNA-binding interface have also been investigated in the ligand-bound state. Such studies relate to the long-standing dilemma between spatial plasticity of transcription factors and their generally high binding affinities, which counteract relocalization in DNA-bound states. In this regard, 'facilitated diffusion' [83] and 'facilitated dissociation' [84] models try to answer how TFs move along DNA, how they pass other bound molecules while diffusing along the double-strands, and how they detach from DNA strands.

On the one hand, facilitated diffusion models describe processes in which the conformational dynamics of a DNA-bound TF can accelerate lateral relocalization along the nucleic acid. E.g., a switch between a tightly DNA-bound conformation and a less strongly bound state can lead to ‘micro dissociation’ [83] that enables dissociative diffusion events that are faster than non-dissociative processes.

**Fig. 2:**
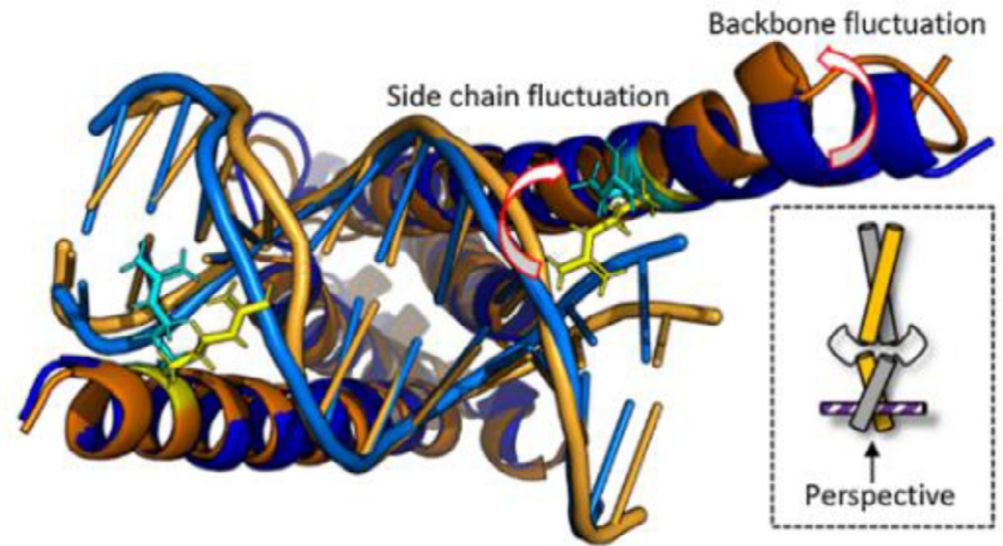
The conformational tuning of the MAX:MAX dimer in the DNA bound state. MD simulations could confirm NMR and EPR data that suggested strong backbone as well as side-chain motions of the DNA-binding site even in the DNA-bound state where the binding sites fold into a stable helical form. The simulations showed how the bound helices open and close continuously around the DNA-strand. (adapted from reference [63] with permission of the publisher.)

To study the structural dynamics involved in facilitated diffusion, experimental observations of PRE data have been reported [61], [63] in combination with ensemble-averaged conformations obtained from MD trajectories. Thus, models of the conformational space of the MAX:MAX-DNA complex could be inferred and it was found that the DNA-binding site undergoes substantial (i.e., spanning several nanometers) structural fluctuations on the nanoseconds timescale that are likely underlying a facilitated diffusion mechanism (Fig. 2) [63]. In particular, opening and closing of the binding domain around the ligand DNA has been reported [63], which was also found for the structurally similar MYC:MAX-DNA complex [85]. It was shown that this conformational tuning underlies an “inchworm stepping”-like motion that facilitates translation along ds-DNA strands. Hence, for both, MAX:MAX- and MYC:MAX-DNA, complexes, comparable modes of diffusion along DNA strands seem to predominate.

On the other hand, facilitated dissociation describes a process in which DNA-bound molecules display elevated rates of dissociation from the double-strand, when other proteins in the surrounding of the TF-DNA complex compete for the nucleic acid. Such processes are important, e.g., when the activity of a transcription factor - which relies on DNA-TF association - needs to be down-regulated [84].

In this regard, Wolynes and co-workers [86], [87] have shown how MD simulations can shed light on facilitated dissociation of the bacterial nucleoid-associated protein Fis. Their results support a ‘three-state’ model that involves the formation of an intermediate ternary complex that facilitates dissociation of the Fis:DNA complex. Besides, the authors revealed by MD simulations that ‘cooperative dissociation’ events can occur upon formation of binary Fis dyads. Here, instead of fostering dissociation of only one TF, both DNA-bound molecules codissociate simultaneously from the DNA – a process reminiscent of “molecular stripping” seen in the NFκB/IκB genetic broadcasting system. [88]

The NF-κB/IκB/DNA genetic switches regulate an extensive array of cellular responses. It has been shown that the IκB factor can actively remove NF-κB molecules from their target DNAs *via* ‘molecular stripping’. This mechanism allows the NF-κB/IκB/DNA switch to function under kinetic control rather than thermodynamic control. [89]

It should be noted that given the strong distance-dependence of PRE effects, the formation of such TF-DNA complexes as well as their facilitated dissociation could complementarily be assessed by means of intermolecular PRE studies. [90] In such an experiment, one interaction partner would be isotopically enriched for NMR detection, while the NMR-silent interaction partner carries the spin-label. Upon complex formation, the proximity between both interaction partners leads to a detectible PRE effect on the NMR-detected molecule, that (i) confirms the computed interaction (and dissociation *vice versa*) and at the same time provides qualitative distance constraints relative to the SDSL site that can complimentarily enlighten the structure of the simulated DNA-protein complex.

*Examples of the combination of NMR and MD Simulations to study TF structure and DNA complex formation*

Next to the use of PREs, a plenitude of studies in the past three years exemplified the potential of integrated magnetic resonance spectroscopy and molecular dynamics simulations. These go beyond the determination of structures from NOE contacts by exploiting the possibility to model conformational features from sparse experimental constraints. In the following, we will present a selection of such approaches to unravel structural and dynamic details of TF-DNA interactions.

Prominently, Afek et al. [91] capitalized on the synergy between NMR and MD simulations integrated with other biomolecular assays to enlighten the impact of DNA mismatches on transcription factor-DNA recognition. In particular, MD results highlighted the effect of the formation of new protein–DNA contacts -- resulting from mismatching -- on binding affinities, while NMR spectroscopy contributed experimental evidence of the DNA base pair geometry.

**Fig. 3:**
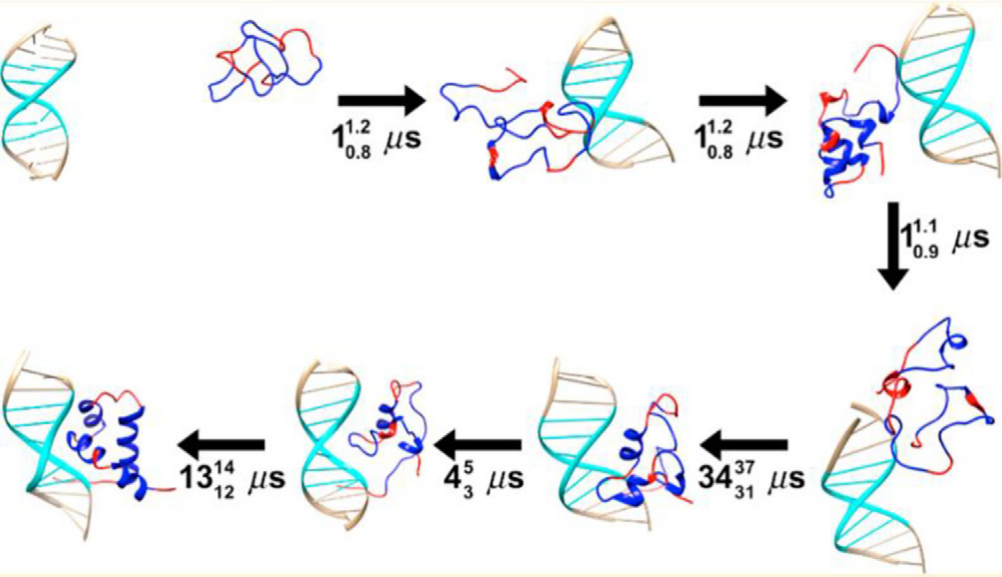
DNA binding of the Drosophila melanogaster transcription factor Brinker (BrkDBD) as an example for induced fit upon binding of TFs to their target DNA. The intrinsically disordered TF recruits the DNA and only folds into a helical state after the encounter complex has been formed. The protein binding region is marked in cyan. The black values indicate the timing required (and confidence bounds) for the various steps. (Adapted from reference [92] with permission of the publisher.) (For interpretation of the references to colour in this figure legend, the reader is referred to the web version of this article.)

Collins and Anderson reported another example [92] (Fig. 3). They showed how the combination of NMR and MD can shed light on the coupled folding and DNA-binding of intrinsically disordered transcription factors. With a 50 μs MD trajectory, they revealed an induced fit-type event in which the target DNA's presence triggers folding of the Drosophila melanogaster transcription factor Brinker (BrkDBD) into its stable bound state.

Along these lines, Dudás et al. showed how to use the NMR/ MD combination to characterize the complex formation between the disordered p53 and the metastasis‐associated factor S100A4. In this case, they reported a so-called 'fuzzy' complex [93], in which the transcription factor does not fold but retains large degrees of intrinsic disorder even in the bound state. [94]

Baired et al. [95] made use of the NMR/MD combination to characterize protein-DNA interactions at hand of lysine side-chain ^15^N-relaxation measurements and comparison to lysine dihedral angles obtained from a molecular dynamics trajectory. Thus, they could quantify the residual conformational freedom of charged lysine amines in TF-DNA complexes.

Besides, not only DNA interactions can be assessed by the combination of NMR and computation. For example, Melikian et al. [96] characterized the binding interface of the NF-κB transcription factor by saturation transfer difference (STD) NMR – highlighting intermolecular contacts -- in combination with computational docking experiments of the TF to a target peptide. Krepl et al. [19] even suggested widely applicable protocols for the combination of NMR and MD to characterize also protein-RNA complexes.

Next to dynamic adaptions upon complex formation and molecular recognition, also structural features of TFs have been analyzed. For example, Escobedo et al. [97] could combine NMR and MD simulations to reveal the functional implications of hydrogens bonds in polyglutamine helices, which are involved in the transcriptional activity of polyglutamine tracts. The authors showed how side-to-main chain contacts stabilize these helices. Maiti et al. [98] studied two HOX transcription factors, SCR and DFD, by comparing spectral density maps from NMR relaxometry -- to describe the internal dynamics of the TF -- with Ramachandran plots obtained from MD simulations -- to highlight the accessible conformational space. The authors could demonstrate that the rigid and flexible segments of these TFs are sequentially conserved to preserve their functions and regulations. Perrez-Borrajero et al. [99], [100] recently described the DNA binding behavior of C-terminal and N-terminal Pax5 paired domains by integrative NMR and dynamic cross-correlation matrices (DCCMs) from MD simulations that describe correlations of backbone motions on a residue basis. Virtanen et al. [101] combined MD simulations and backbone ^15^N-spin relaxation data to derive structural ensembles of the Engrailed 2 transcription factor. Further, Barros et al. [102] compared the wild-type of tumor suppressor p53 with a Y220C mutant, employing Markov state models (MSM) and time-lagged independent components analysis (tICA). Thus, they revealed a formerly unknown allosteric feedback between two flexible loops. Here, the use of MSM enabled the observation of slow motional modes (µs-ms) that were validated by NMR relaxometry.

## 3 Combining EPR and MD simulations

When a transcription factor or its target DNA carries a paramagnetic moiety, e.g., a spin-label or a metal center as used in PRE-NMR, direct detection of the electron spin by electron paramagnetic resonance (EPR) spectroscopy can provide valuable information complementary to NMR data [103], [104].

A compelling approach makes use of doubly spin-labeled proteins for nanoscale distance measurements; a method which is often denoted as DEER (double electron–electron resonance) or PELDOR (pulsed electron double resonance) spectroscopy [36], [40], [41]. With this method, it is possible to experimentally determine the distribution *P*(*r*) of distances *r* between two SL attached to selected sites of the target molecule. The same distance distribution can be computed *in-silico* through the extraction of atom-to-atom distances from molecular dynamics trajectories [105], [106]. The two distributions can be quantitatively compared to confirm experimental results and provide 3D models that ease the interpretation of spectroscopic data.

**Fig. 4:**
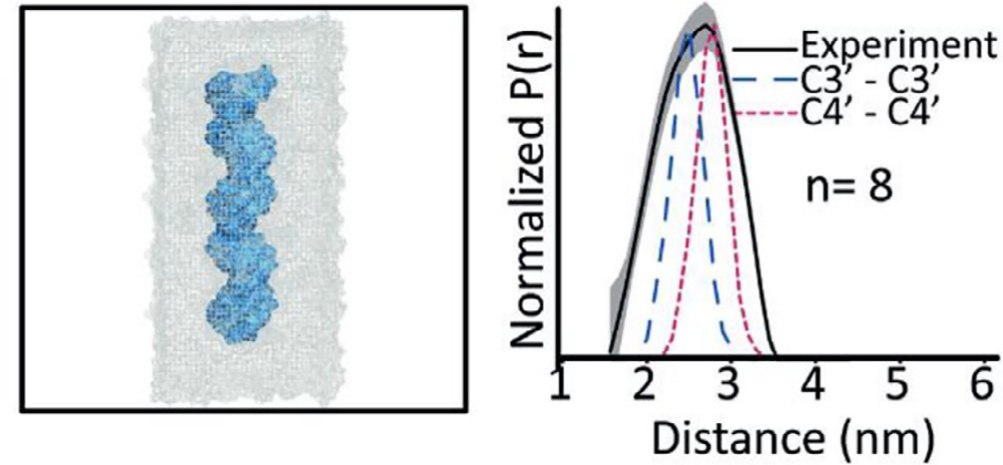
Analysis of DNA conformation by EPR and MD. a) Snapshot of the simulated DNA molecule in a water box used to predict the experimental distance distributions. b) The experimental distance distribution P(r) (black) obtained by DEER-EPR using Cu(II)–based spin labels could be reproduced by molecular dynamic simulations (red and blue). Reproduced from reference [107] with the permission of the publisher.) (For interpretation of the references to colour in this figure legend, the reader is referred to the web version of this article.)

Concerning nucleic acid-interactions, an approach that complements tagging of proteins is to label oligonucleotides doubly. Such approaches can reveal, e.g., DNA structure fluctuations[107], switches between various RNA conformations [108] that occur on the nanoseconds timescale (see Fig. 4) or the orientation of DNA helices [109].

Another widespread tool to assess DEER-EPR data through computational analysis is based on pre-computed rotamer libraries of spin-labels, which can complement the computation of entire MD trajectories and speed up data assessment [110], [111], [112]. With this method, it is possible to predict the distance distribution between two spin-labels within seconds via projections of long MD trajectories of spin labels -- attached *in-silico* to a protein model -- onto sparse sets of dihedral angles. The resulting prediction can then be compared with experimental distance distributions.

**Fig. 5:**
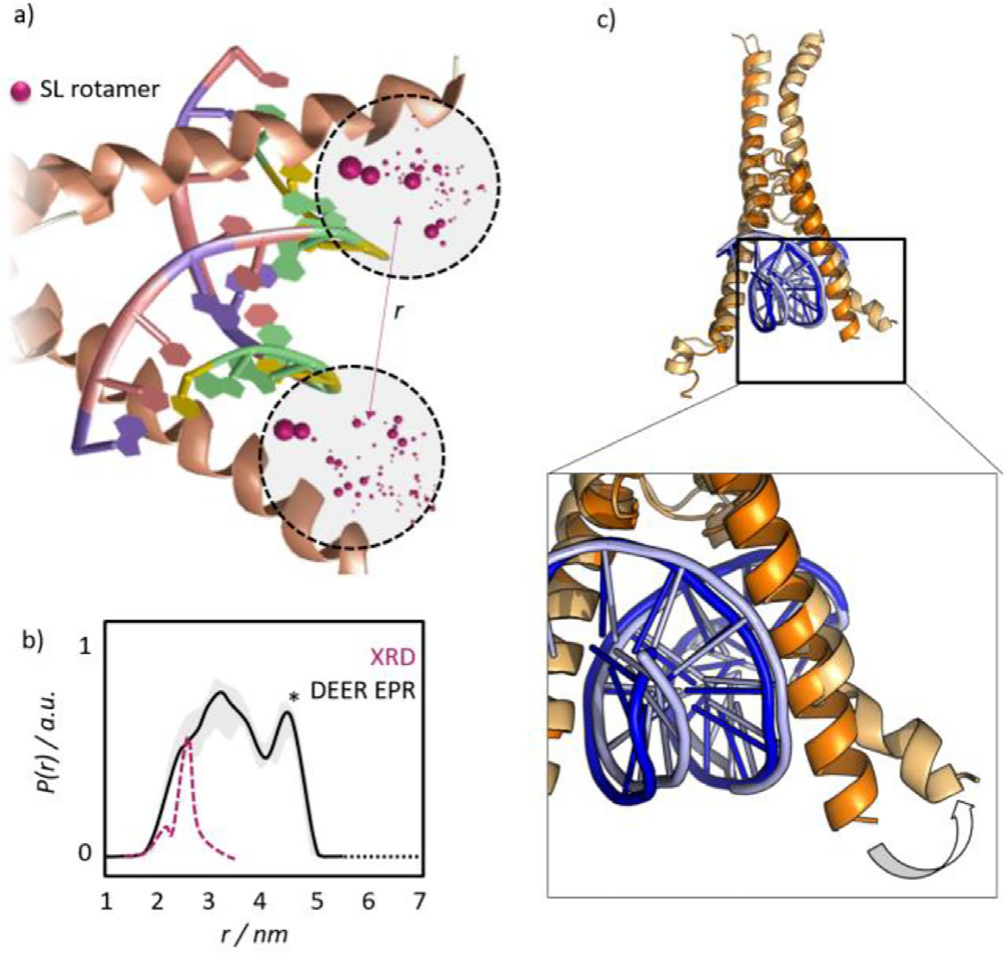
Assessment of TF conformations in their DNA-bound state by EPR nanoscale distance measurements. a) Rotamer distributions predicted for two spin labels attached to the DNA-binding domain of MAX:MAX. The purple dots indicate the conformational freedom of the nitroxide (MTSL) labels attached to the transcription factor. b) The experimental distance distribution obtained by EPR (black) compared to the distribution computed from the crystal structure through a rotamer analysis (purple). Only the most compact state is represented by the XRD-derived structure, while a broader conformational ensemble is found in solution by EPR. c) The conformational sampling of DNA-bound MAX:MAX found in MD simulations confirmed that the DNA-binding domain opens and closes continuously around the bound DNA-ligand. This conformational tuning results in the broad experimental distance distribution shown in panel b. (adapted from reference [63] with permission of the publisher.) (For interpretation of the references to colour in this figure legend, the reader is referred to the web version of this article.)

Employing this approach, the crystal structure [113] of DNA-bound MAX:MAX was recently found to represent only the energetic minimum of a large and heterogeneous conformational ensemble that the TF-DNA complex samples in solution (Fig. 5) [63].

Due to the long-range character of electron–electron dipolar couplings, nanoscale distance measurements by EPR are particularly well-suited to assess such large-scale conformational fluctuations that are suppressed in the crystalline state (Fig. 5). In the light of the above-mentioned facilitated diffusion along DNA-strands, the assessment of such dynamics that take place on length scales of several nanometers appears particularly relevant.

## 4 Methodological considerations

When studying intrinsically disordered proteins or domains, each of the here reviewed methods faces its particular challenges. In the following, we comment on the scope, complementarity, and limitations of the three methods, NMR, EPR, and MD simulations.

*NMR and MD*: In NMR studies of IDPs and IDRs, an often encountered challenge lies in the absence of any three-dimensional protein model. This results in abstractness of the acquired data and thus complicates their interpretation. In such cases, relative changes in residue-specific structural dynamics are typically acquired and compared between different protein sites [12]. For example, more flexible and rigid regions along the primary sequence can be localized using relaxation parameters, or statements about qualitative distance constraints can be made from PRE experiments, e.g., identifying distant and proximate residues relative to a labeling site [66]. However, the association of such information with residue-resolved structural models remains often beyond the scope of stand-alone NMR studies of disordered proteins.

The combination with simulated structures can help to overcome this limitation. By cross-validating the NMR-derived information with computed MD trajectories, the computed model can (i) be confirmed, mitigating the impact of finite-length effects on MD simulations of IDPs and IDRs; and (ii) in return, the NMR-derived data can be interpreted at hand of these models. This complementarity eases data interpretation by providing insights into the structural features that underlie the otherwise quite abstract spectroscopic parameters.

*EPR and MD:* A similar situation arises for EPR spectroscopy. For example, the distance constraints obtained by DEER experiments can be hard to interpret in the absence of any structural model describing the investigated molecule. Hence, the comparison with computed structures can also here assist the experimental data interpretation. In particular, nanoscale distance distributions are readily extractable from MD trajectories and can be compared with experimental DEER data.

*NMR and EPR:* Compared to PRE NMR, distance distributions obtained from DEER are often quantitative [114] but only represent constraints specific to the chosen SDSL sites. In contrast, PREs are often interpreted qualitatively, yet residue-resolved PRE data are acquired regularly for IDPs and IDRs. Hence, between NMR and EPR of spin-labeled molecules, another aspect of complementarity arises. While NMR provides a comprehensive overview of the residue-specific conformational dynamics of a protein or nucleic acid, EPR can complement this information with quantitative distance constraints.

*MD and magnetic resonance:* MD simulations of intrinsically disordered biomacromolecules often face the question, which fraction of the conformational space is represented by the computed trajectories. In particular, for large IDPs and IDRs, it is often complicated to derive faithful representations of structural ensembles from MD simulations alone. The conformational space that needs to be sampled is so vast that the computational costs for simulation of the entire sampling space can become exceedingly high. Here cross-validation versus experimental data can help to ascertain the representatively of the computed ensemble [115]. In other words, if the computed data enables a satisfactory reproduction of the experimental data, the derived structures likely represent a significant share of the conformations sampled by the molecule.

Importantly, recent years have witnessed the development of maximum entropy (and recently also maximum parsimony principles) that can greatly assist the integration of computed and experimental data [60], [116], [117]. These approaches rely on the principle that the optimal probability distribution of calculated states (conformations) that agree with the experiment data displays a maximized Shannon entropy [118]. Hence, the general idea is to overcome potential inaccuracies of the computed models and finite sampling effects, which often result divergent simulated and computed results. In maximum entropy and maximum parsimony approaches, the experimental data is therefore employed to refine the MD simulations so that the predicted conformational ensemble matches the experimental constraints.

*Choosing a method:* The choice of the employed combination of methods for investigating a particular system depends on many factors. These are often too complex to be assessed *a priori*. However, a few statements can be made that might be helpful in future applications.

NMR spectroscopy is well-suited for investigations of IDPs and IDRs with less than (typically) 400–500 residues. In such cases, the resolution and signal intensity that can be achieved with a state-of-the-art spectrometer suffices to obtain information about residue-resolved structural dynamics. Besides, NMR can be well combined with MD simulations when the molecular weights of the protein-DNA complexes are not too high (typically less than 0.1 MDa [119]). For heavier complexes signal amplitudes often become prohibitively weak due to fast nuclear relaxation.

In contrast, EPR is not size limited, which enables studies of larger complexes and TFs. The long distances that can be assessed by DEER (>10 nm in favorable cases) render this choice particularly advantageous. Besides, as only specific sites are selected for SDSL, EPR is also a helpful tool for studying DNA double strands. Here NMR spectroscopy is impeded by strong signal overlap as the resonances of the different nucleotides tend to resonate at similar frequencies.

## 5 Outlook

The growing power of modern computers enables access to ever-longer and precise molecular dynamics trajectories. This development bears the potential to support experimental methods for structural characterization with three-dimensional models that ease data interpretation.

Especially for magnetic resonance techniques, where data interpretation can become quite abstract complementary approaches are often fruitful. In this regard, applications to transcription factors appear to be popular as long-standing questions can be revisited with new integrative approaches, e.g., the dilemma between low DNA-TF dissociation constants and spatial as well as functional plasticity.

We expect the combination of NMR, EPR and MD to become increasingly valuable, as structural ensembles of intrinsically disordered transcription factors can be characterized on a residue basis. Indeed, NMR alone could not provide three-dimensional models of such dynamic systems in the absence of NOE constraints, while computational data still require experimental confirmation considering the vast conformational spaces sampled by IDPs and IDRs.

In particular, for practical applications, it should be considered that NMR spectroscopy can contribute residue-resolved structural dynamics of a biomacromolecule, while complementarily, EPR can provide quantitative distance constraints between well-defined mutation sites. Both pieces of information can be well integrated with MD data resulting in advantageous complementarity between the three methods.

The challenge in the simulation of complex biomolecules often lies in the computation of the conformational ensemble's representative fractions. Typically, to provide feasible data sets, simulations over (at least) several hundredths of nanoseconds are necessary together with a suitable number of replica runs. Hence, depending on the size of the simulated IDR, such calculations can become very expensive such that faster computers are valuable.

In return, NMR spectroscopy is limited by the sizes of the proteins and nucleic acids under study, which often determine the quality of the spectra. For large macromolecules, resonances are often broadened beyond detection, and in addition, signal overlap in crowded spectra can impede data interpretation. Currently, studies of biomolecules with typically less than 400–500 residues are feasible with conventional approaches. In this regard, the development of spectrometers with ^1^H resonance frequencies of 1 and 1.2 GHz can help to push the size limits of the state-of-the-art NMR [120].

Concerning EPR spectroscopy, a major bottleneck is the introduction of the spin-label that can significantly bias the structure and function of a protein or nucleic acid. The design and use of spin-labels with minimal structural impact [121], [122] is a promising step to alleviate this circumstance.

## CRediT authorship contribution statement

**Fanny Kozak:** Writing - original draft, Visualization, Writing - review & editing. **Dennis Kurzbach:** Supervision, Writing - review & editing.

## Declaration of Competing Interest

The authors declare that they have no known competing financial interests or personal relationships that could have appeared to influence the work reported in this paper.
